# Laboratory IR Spectra of the Ionic Oxidized Fullerenes C_60_O^+^ and C_60_OH^+^

**DOI:** 10.1021/acs.jpca.2c01329

**Published:** 2022-05-09

**Authors:** Julianna Palotás, Jonathan Martens, Giel Berden, Jos Oomens

**Affiliations:** † Institute for Molecules and Materials, FELIX Laboratory, Radboud University, Toernooiveld 7, 6525ED Nijmegen, The Netherlands; ‡ van ’t Hoff Institute for Molecular Sciences, University of Amsterdam, Science Park 904, 1098XH Amsterdam, The Netherlands

## Abstract

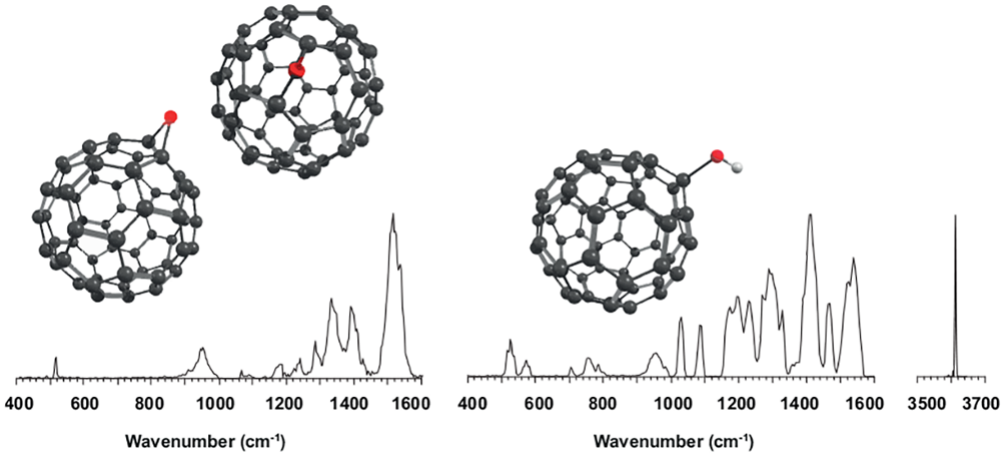

We present the first experimental vibrational spectra of gaseous oxidized derivatives of C_60_ in protonated and radical cation forms, obtained through infrared multiple-photon dissociation spectroscopy using the FELIX free-electron laser. Neutral C_60_O has two nearly iso-energetic isomers: the epoxide isomer in which the O atom bridges a CC bond that connects two six-membered rings and the annulene isomer in which the O atom inserts into a CC bond connecting a five- and a six-membered ring. To determine the isomer formed for C_60_O^+^ in our experiment—a question that cannot be confidently answered on the basis of the DFT-computed stabilities alone—we compare our experimental IR spectra to vibrational spectra predicted by DFT calculations. We conclude that the annulene-like isomer is formed in our experiment. For C_60_OH^+^, a strong OH stretch vibration observed in the 3 μm range of the spectrum immediately reveals its structure as C_60_ with a hydroxyl group attached, which is further confirmed by the spectrum in the 400–1600 cm^–1^ range. We compare the experimental spectra of C_60_O^+^ and C_60_OH^+^ to the astronomical IR emission spectrum of a fullerene-rich planetary nebula and discuss their astrophysical relevance.

## Introduction

In a series of experiments aimed to explore the chemistry in interstellar and circumstellar space, Kroto, Smalley, and Curl discovered the iconic C_60_ molecule.^1^ Ever since, fullerenes have attracted much attention, not only within astrochemistry but also within the physics, chemistry, and material sciences disciplines.^2,3^ As a consequence, C_60_ has been characterized very thoroughly, including its ion chemistry and spectroscopic properties,^4−8^ which are especially valuable in the quest for the astronomical abundance of this molecule and several of its analogues. On the basis of their IR and near-IR spectroscopic features, fullerenes have been identified in various parts of interstellar and circumstellar space.^9−13^ Furthermore, C_60_^+^ has been associated with two of the diffuse interstellar bands (DIBs) at 9633 and 9578 Å using accurate cold gas-phase laboratory experiments.^4^

With the occurrence of the C_60_ and C_70_ fullerenes in circumstellar environments firmly established, the quest continues for fullerene derivatives that may also be detectable. Numerous derivatives have been suggested to occur, where modifications involving elements of high cosmic abundance are obviously strong candidates. Protonated, hydrogenated, and oxidized fullerenes have therefore often been hypothesized (also by Kroto himself^14^), as well as some metallofullerenes.^15^ Laboratory investigations aiming to support astronomical searches have indeed been reported for hydrogenated^16,17^ and protonated^18−20^ fullerenes.

In this paper, we focus on oxidized fullerene derivatives, which have often been regarded in an astrophysical context. It has been shown that under γ-irradiation in water ices, C_60_ became soluble as a result of hydroxylation and oxidation processes.^21^ An example of an astrophysical environment that may be of particular interest for oxidized hydrocarbon and fullerene species is the binary star HD 44179, also called the Red Rectangle, that exhibits C-rich but also O-rich regions. The intersections between those phases are possibly environments for exciting chemical processes and molecules containing carbonyl groups have been suggested as possible carriers of the emission features in the Red Rectangle.^22^ Among other exohedral complexes of buckminsterfullerene, C_60_O^+^ was suggested as a carrier of the features observed on top of the continuum red emission.^23^ To interpret the observed data and understand the chemical processes, laboratory investigation of oxidized carbonaceous molecules is necessary.

**Figure 1:**
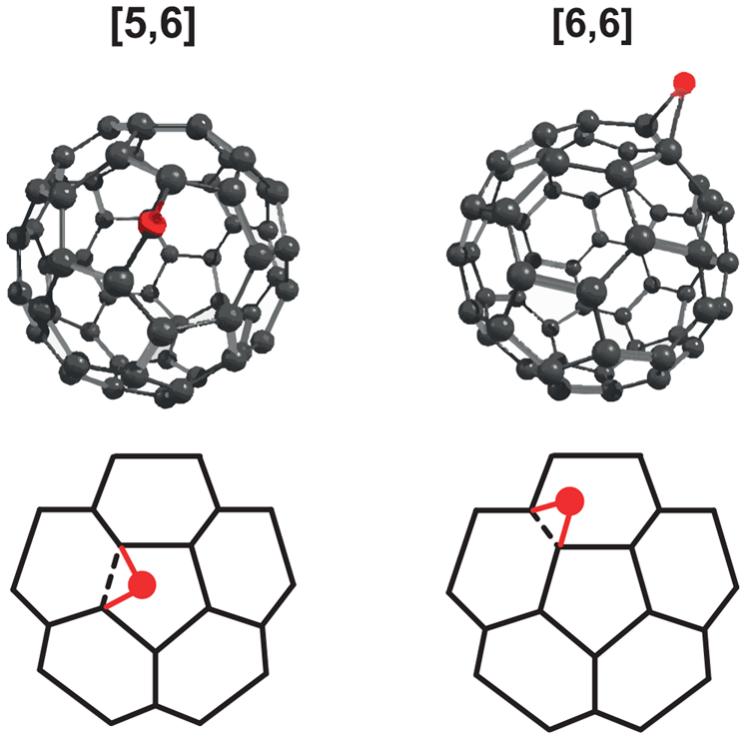
[5,6] and [6,6] isomers of C_60_O.

C_60_O, as the simplest fullerene oxide, serves as an ingredient in synthesis processes^2,24−26^ and has been the subject of many studies investigating its stability and structure.^27−29^ Addition of atomic oxygen to the carbon cage can be hypothesized to lead to different isomeric configurations of C_60_O. The two most prominent isomers are shown in Figure 1: in the epoxide form [6,6], the oxygen bridges two C-atoms that connect two six-membered rings, whereas in the annulene-like form [5,6], the oxygen inserts into a CC bond connecting a five- and a six-membered ring. CC bond epoxidation versus insertion yield clearly distinct values for COC angles and CC distances (see Table 1). In addition, the two C_60_O isomers have different symmetries: the epoxide form [6,6] belongs to the *C*_2*v*_ point group and the annulene-like form [5,6] to the *C*_*s*_ point group. Early theoretical studies based on semiempirical and relatively low-level ab initio quantum-chemical calculations determined that the [5,6] structure is the most stable isomer.^30−33^ However, more recent theoretical investigations at the density functional theory (DFT) level have revealed a strong basis set dependence of the relative energies of the two isomers.^34^ The energy difference between the two isomers decreases and reverses sign for larger basis sets, suggesting that the [6,6] isomer is actually more stable. The energetic barrier connecting the two isomers is significant, 226 kJ/mol above the [6,6] epoxide minimum-energy structure.^34^ Experimental studies show that both configurations are possible depending on the synthesis.^35^ The annulene-like form can dimerize or convert into the epoxide structure after irradiation,^36^ suggesting that the epoxide is indeed lowest in energy.

**Table 1:** Selected Bond Angles and Distances at the Optimized Geometries of C_60_O^+^ and C_60_O^a^

		C–C distance (Å)	C–O distance (Å)	C–O–C angle (deg)
C_60_O^+^	[5,6]	2.17	1.37	104.6
	[6,6]	1.53	1.41	65.2
C_60_O	[5,6]	2.15	1.38	101.9
	[6,6]	1.53	1.42	65.5

a Values are computed at the B3LYP/6-311+G(d,p) level of theory.

IR spectra have been reported for neutral C_60_O and fullerenes with hydroxyl groups have also been studied.^37^ To the best of our knowledge, the structure of fullerene oxide in ionic form has not been studied to date. Here we present gas-phase IR spectra of C_60_O^+^ and C_60_OH^+^.

## Methods

The gas-phase infrared multiple-photon dissociation (IRMPD) spectrum of C_60_OH^+^ and C_60_O^+^ ions were measured in a modified 3-D quadrupole ion trap (QIT, Bruker amaZon Speed ETD) coupled to the Free-Electron Laser for Infrared eXperiments, FELIX.^38,39^

### Ionization

Ions are produced in an atmospheric pressure chemical ionization (APCI, Bruker APCI II) source using a direct insertion probe (DIP, Bruker DP) assembly. The APCI source is particularly suitable for studying apolar substances such as PAHs^40^ and fullerenes.^19,20^ The DIP inlet replaces the spray nebulizer on the standard Bruker APCI ion source. The solid sample is placed on the tip of a single-use glass tube of the DIP, where it is heated and sublimates into the APCI source chamber. Ionization occurs in the plasma of the corona discharge. The DIP-APCI source enables especially the study of molecules with low solubility and moreover features a minimal sample consumption.^40^

In the present experiments, small amounts of C_60_ (MER Corporation, USA) are placed on the glass tip and then introduced into the APCI source. In addition to the radical cation and protonated form of C_60_ at *m*/*z*-values of 720 and 721, respectively, the source produces ions at *m*/*z* 736 and 737, corresponding to oxidized C_60_ in its radical cation (C_60_O^+^) and protonated (C_60_OH^+^) form, presumably as a consequence of water vapor and/or O_2_ present in the APCI source region.

The ion intensity ratio between the protonated and radical cation C_60_O depends on the temperature setting of the APCI vaporizer. Setting the heater temperature to 370 °C gives a strong signal for the protonated species, whereas at 450 °C the radical cation has a high ion count as well. The sample vapor is carried along by N_2_ gas at 1 bar. The corona current is 4 μA and the bias voltage between the spray shield and capillary cap is set to 500 V. The potential of the capillary cap is set to −4500 V, relative to the grounded heater assembly.

**Figure 2:**
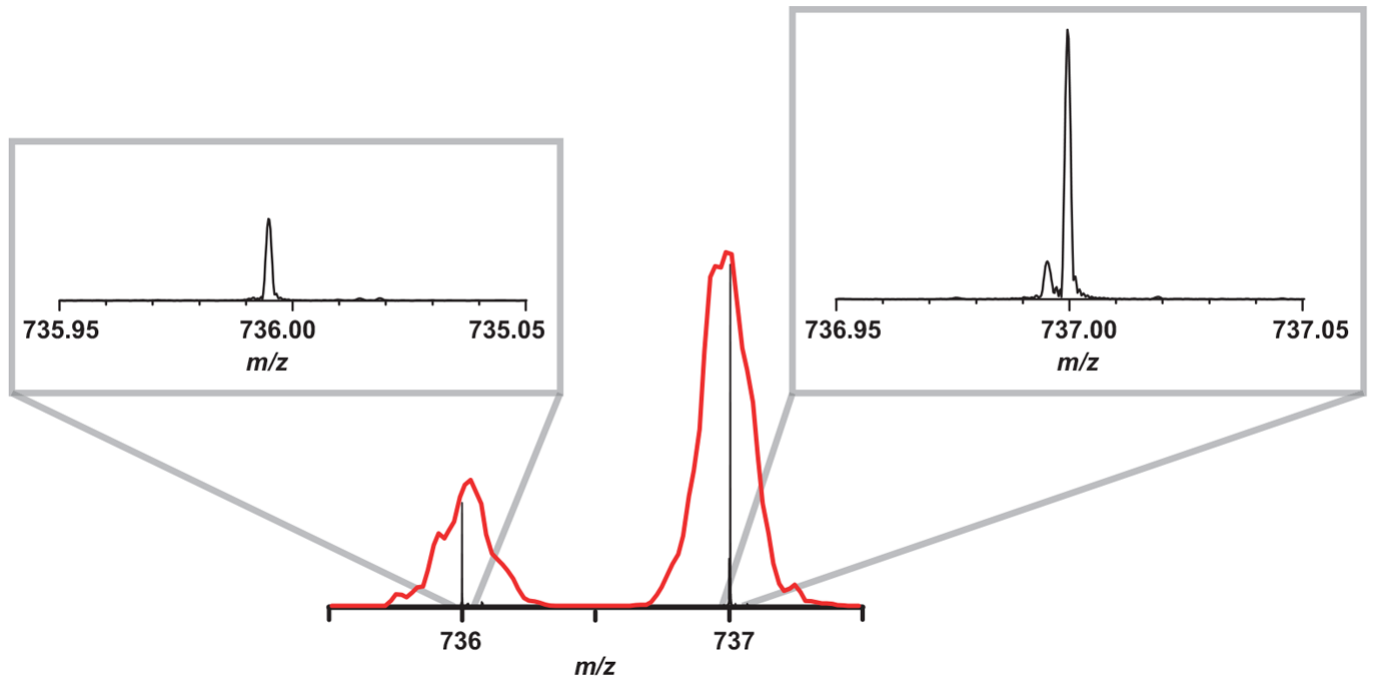
High-resolution mass spectrum of the fullerene sample as generated using the APCI source and recorded with a Fourier-transform ion cyclotron resonance spectrometer. On the top left figure, the *m*/*z* 736 peak corresponds to ^12^C_60_O^+^. On the top right, the *m*/*z* 737 peak has two components: the minor ^13^C^12^C_59_O^+^ and the major ^12^C_60_OH^+^ peak. The IRMPD spectra are recorded in a QIT MS, which is unable to resolve the two ions. For comparison, the high-resolution mass spectrum (black) is overlapped with the mass spectrum recorded in the QIT MS (red) on the bottom figure.

Because of the natural abundance of ^13^C, the mass peak at 737 is a superposition of two ions: ^13^C^12^C_59_O^+^ and ^12^C_60_OH^+^. Mounting the DIP-APCI source on a Fourier-Transform Ion Cyclotron Resonance mass spectrometer (FTICR-MS, Bruker solariX XR), we are able to resolve the two ions, as shown in the high-resolution mass spectrum in Figure 2, which has been recorded at a heater temperature of 370 °C. We establish that 12% of the ion signal at a nominal mass of *m*/*z* 737 is because of ^13^C^12^C_59_O^+^. The ion trap mass spectrometer used for the IR spectroscopy measurements cannot resolve the small mass difference of the two *m*/*z* 737 ions.

### IRMPD Spectroscopy

To record IR spectra, we isolate the ions at the nominal mass of interest, that is, *m*/*z* 737 for C_60_OH^+^ and 736 for C_60_O^+^. The trapped ion cloud is then irradiated with the tunable IR laser light from FELIX. IR-induced dissociation of the *m*/*z* 736 peak leads to fragmentation into the *m*/*z* 720 channel, indicating loss of atomic oxygen. As is shown in Figure 2, the *m*/*z* 737 ion is a superposition of protonated C_60_O and the ^13^C containing radical cation of C_60_O. We observe two dissociation channels, *m*/*z* 721 and 720. The ^13^C^12^C_59_O^+^ ions fragment only to *m*/*z* 721 via expulsion of an O atom. The C_60_OH^+^ ion may hypothetically dissociate via either loss of an OH radical or loss of atomic oxygen.
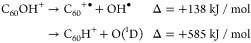
1The reaction forming ground state O(^3^P) is spin forbidden and therefore ignored here. Clearly, loss of an OH radical is thermodynamically favored by a large margin (even if it breaks the even-electron rule in mass spectrometry). Therefore, we assume that only OH loss is detected from the C_60_OH^+^ ions and that *m*/*z* 720 is the fragment channel of interest that we monitor to obtain the IRMPD spectrum of C_60_OH^+^; possible contributions from ^13^C^12^C_59_O^+^ to the spectrum are therefore excluded.

After irradiation, a mass spectrum of the ions in the trap is recorded. Six mass spectra are averaged at each wavelength, after which the frequency is tuned in steps of 3 cm^–1^ and the whole MS sequence is repeated. The wavelength-dependent fragmentation of the ions is expressed as a fragmentation yield via
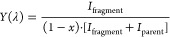
2where *x* is the fraction of the ^13^C radical cation contributing to the peak intensity of the protonated ion at the same *m*/*z*. This fraction is 0.12 for C_60_OH^+^ and 0 for C_60_O^+^. The fragment fluence *S* is then obtained as^41^
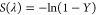
3*S*(λ) was linearly corrected for variations of the laser pulse energy over the scan range and for the irradiation time.

In the 10–25 μm region, the He buffer gas pressure in the quadrupole ion trap was reduced to its minimum value to reduce collisional quenching of the IR-excited ions. Although the lower He pressure leads to a lower ion count, IR-induced fragmentation is more efficient so that we can detect low-intensity peaks in the IRMPD spectrum. In addition, CsI windows were used on the vacuum housing of the ion trap in the 10–25 μm region that allowed us to measure low-intensity bands in the spectra. As compared to the commonly used KRS-5 windows, CsI has a better IR transparency, but it cannot be used below 10 μm because of the lower damage threshold.

FELIX produces macropulses at a 10 Hz repetition rate that are about 7 μs long and that consist of a train of micropulses spaced by 1 ns. The micropulses are Fourier-transform limited and have a bandwidth of 0.4% of the IR frequency. The IR spectrum was recorded between 6 and 25 μm, and in this wavelength region, FELIX produces macropulse energies up to approximately 150 mJ. The wavelength is calibrated with a grating spectrometer with an accuracy of ±0.01 μm.

In the 3 μm region, the same experiment is carried out with an optical parametric oscillator (OPO) pumped by a 80 MHz picosecond fiber laser.^42^ The OPO pulses have a duration of 35 ps and a bandwidth of 0.5 cm^–1^. The IR frequency is tuned between 3500 and 3700 cm^–1^, where the output power of the OPO is around 400 mW. In these measurements, the trapped ions are irradiated for 50 ms. The IR frequency was calibrated by recording the IR spectrum of protonated tryptophan (*m*/*z* 205) using the strongest vibrational band at 3555 cm^–1^.^43^

### Quantum-Chemical Computations

Quantum-chemical calculations were performed at the density functional theory (DFT) level using different combinations of functionals and basis sets. Geometry optimizations were performed using the B3LYP hybrid functional with 4-31G and 6-311+G(d,p) basis sets. In addition, the B3PW91 hybrid functional was applied with the 6-311+G(d,p) basis set to test the effect of a higher level of electron correlation as was previously suggested by ref (34). We tested the dispersion-corrected M06-2X functional with the 6-311+G(d,p) basis set, as well. For each optimization, a vibrational analysis (within the harmonic oscillator approximation) was performed at the same level of theory to verify that the geometries corresponded to true minima and to derive relative Gibbs free energies of the two isomers.

For comparison with experimental IRMPD spectra, the vibrational spectra computed at the B3LYP/6-311+G(d,p) level were employed. Computed frequencies were scaled by 0.967 for the frequency range below 2000 cm^–1^. For the protonated system, the OH-stretch vibration was scaled with a factor of 0.955 that is often used in the 3 μm range of vibrational spectra.^44^ Calculated stick spectra were convolved with a Gaussian line profile with full width at half-maximum (fwhm) of 1% of the wavenumber. All calculations used the Gaussian16 software package at the Dutch national supercomputer Cartesius at SURFsara.

## Results and Discussion

### C_60_O^+^

As outlined in the Introduction, considering only the exohedral oxygen attachment, neutral C_60_O has two stable isomers. The oxygen atom either can bridge two carbon atoms that fuse two six-membered rings forming an epoxide moiety ([6,6] isomer) or can insert into a CC bond that fuses a five- and a six-membered ring, which is referred to as an annulene-like (open) structure ([5,6] isomer). In Table 1, the bond lengths and bond angles of these configurations are listed.

An earlier study for neutral C_60_O found that high-level computations place the [6,6] isomer lower in energy, although the difference with the [5,6] isomer is marginal and reversed stabilities are found depending on the basis set size and the level of electron correlation incorporated.^34^ Nonetheless, experimental observations, including the IR spectrum at 10 K,^31^ appear to confirm that the [6,6] isomer is indeed the global minimum structure.

Here we repeat the calculations for the two isomers of C_60_O and include an evaluation of the two isomers in their radical cation form, C_60_O^+^. In Table 2, the relative Gibbs energies obtained at different levels of theory are summarized. At the B3LYP/6-311+G(d,p) level of theory, the neutral [5,6] isomer is slightly lower in energy (−7 kJ/mol), in line with values in ref. (34) using a slightly smaller basis set. Also in agreement with this previous study, the gap between the two isomers decreases as the basis set size increases and stabilities reverse using the B3PW91 functional. In addition, the M06-2X functional predicts the same reversal of stabilities and suggests the [6,6] isomer to be the global minimum.

**Table 2:** Gibbs Energies of the Isomers of C_60_O^+^ and Neutral C_60_O Using Different Combinations of Functionals and Basis Sets^a^

		B3LYP		B3PW91	M06-2X
		4-31G	6-311+G(d,p)	6-311+G(d,p)	6-311+G(d,p)
C_60_O^+^	[5,6]	0	0	0.3	0
	[6,6]	27.7	12.3	0	5.9
C_60_O	[5,6]	0	0	5.4	3.4
	[6,6]	21.7	7.2	0	0

a The relative values listed here are in kJ/mol.

In the radical cation, the [5,6] isomer appears to be more stabilized using the B3LYP functional. At the B3PW91/6-311+G(d,p) level, the two isomers are now iso-energetic. M06-2X places the [5,6] isomer below [6,6] by a margin of 6 kJ/mol, but overall, it is fair to say that is not possible to decide which of the two isomers is preferred on the basis of computational results alone.

**Figure 3:**
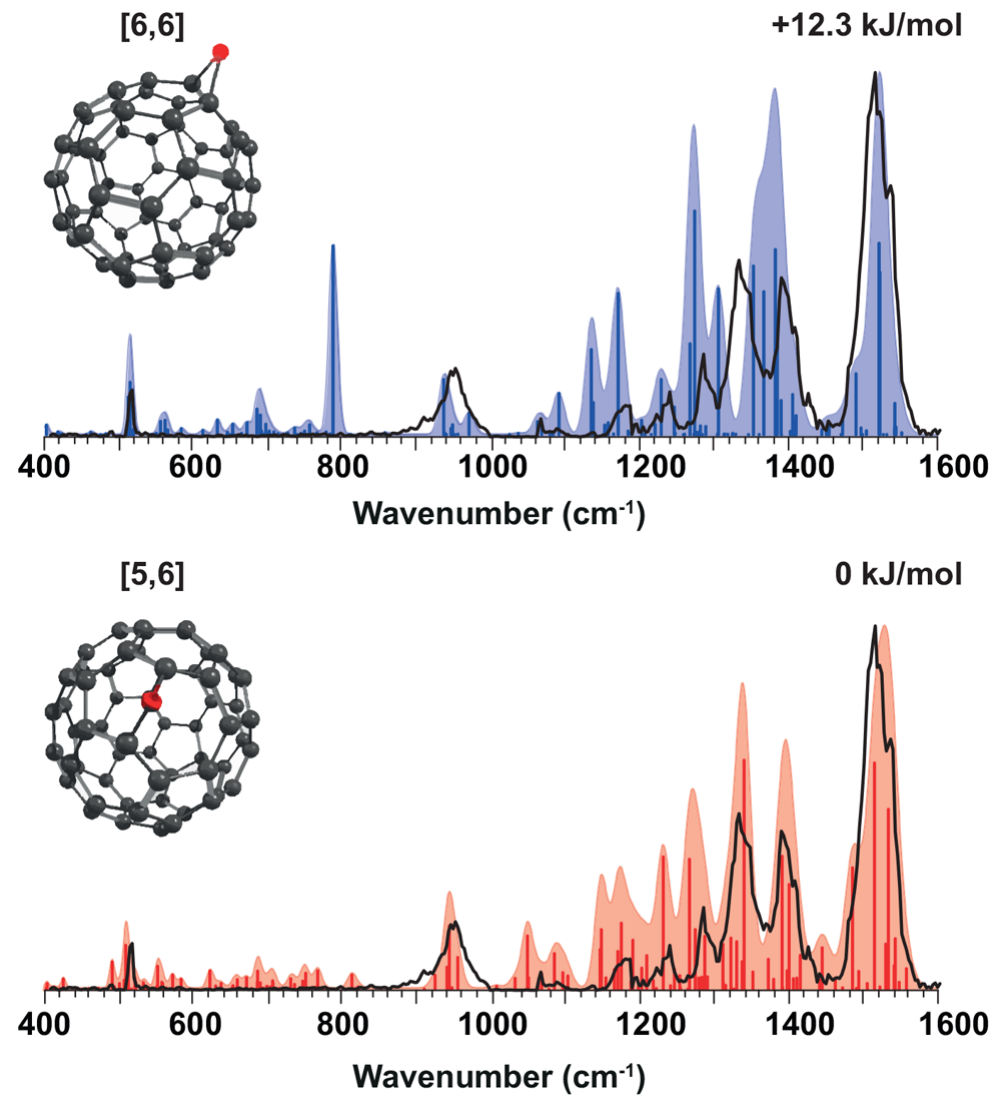
IRMPD spectrum of C_60_O^+^ (black line) compared to theoretical spectra of the two isomers, the [5,6] (red) and [6,6] (blue) configurations. The computed IR spectra are calculated at the B3LYP/6-311+G(d,p) level, and a frequency scaling factor of 0.967 is used.

Figure 3 shows the IRMPD spectrum recorded for the C_60_O^+^ radical cation. The experimental spectrum is compared with the calculated IR spectra of both isomers. The figure shows the spectra produced at the B3LYP/6-311+G(d,p) level of theory, which has been shown to reliably predict IR spectra, including those of (charged) fullerenes.^19,20^ A qualitative visual inspection suggests that the predicted spectrum for the open form [5,6] matches the experimental spectrum better. In this assignment, a few bands play a particularly diagnostic role. The experimental band centered at 1333 cm^–1^ is poorly reproduced by the [6,6] theoretical spectrum and one of its strongest predicted bands falls between two distinct experimental bands at 1333 and 1389 cm^–1^. Moreover, the [6,6] isomer is predicted to have a strong band just below 800 cm^–1^ that is absent in the experimental spectrum; note that weaker predicted bands in this far-IR range are actually observed, and only the much weaker bands predicted between 550 and 800 cm^–1^ escape detection. In contrast, the diagnostic bands at 1333 and 1389 cm^–1^ and the absence of a band near at 800 cm^–1^ are favorably reproduced by the [5,6] computed spectrum, especially if we ignore deviations in relative intensity, which we attribute to nonlinearities in IRMPD, subtle effects of spectral convolution and the limited accuracy of the computed intensities.

Hence, despite the very similar computed energetics, the IR spectral analysis suggests that the [5,6] isomer is formed in our experiment. A contribution from the [6,6] isomer can obviously not be excluded entirely, although its fraction ought to be minor on the basis of the absence of experimental intensity near 800 cm^–1^; a Boltzmann distribution of the two isomers at 293 K would give 9% of the [6,6] isomer taking the M06-2X energy difference. The band centers extracted from the computed spectra after convolution with a Gaussian line shape function are listed in Table S1. The root-mean-square deviations from the experimental band centers are given at the bottom of the table. These RMS deviations in band positions further support the conclusion that the [5,6] isomer of C_60_O^+^ is predominantly formed in the APCI source.

In addition, we explored the potential energy surface (PES) of the C_60_O^+^ with the B3LYP functional and a smaller basis set (4-31G). There is one transition state (TS) connecting the two isomers, where the oxygen is roughly directly above a single carbon atom. The TS lies 127 kJ/mol higher in energy than the [5,6] minimum structure. This value is lower than the TS in neutral C_60_O computed by Sohn et al.^34^ but high enough to suggest that the C_60_O^+^ ion has two well-separated isomers.

### C_60_OH^+^

**Figure 4:**
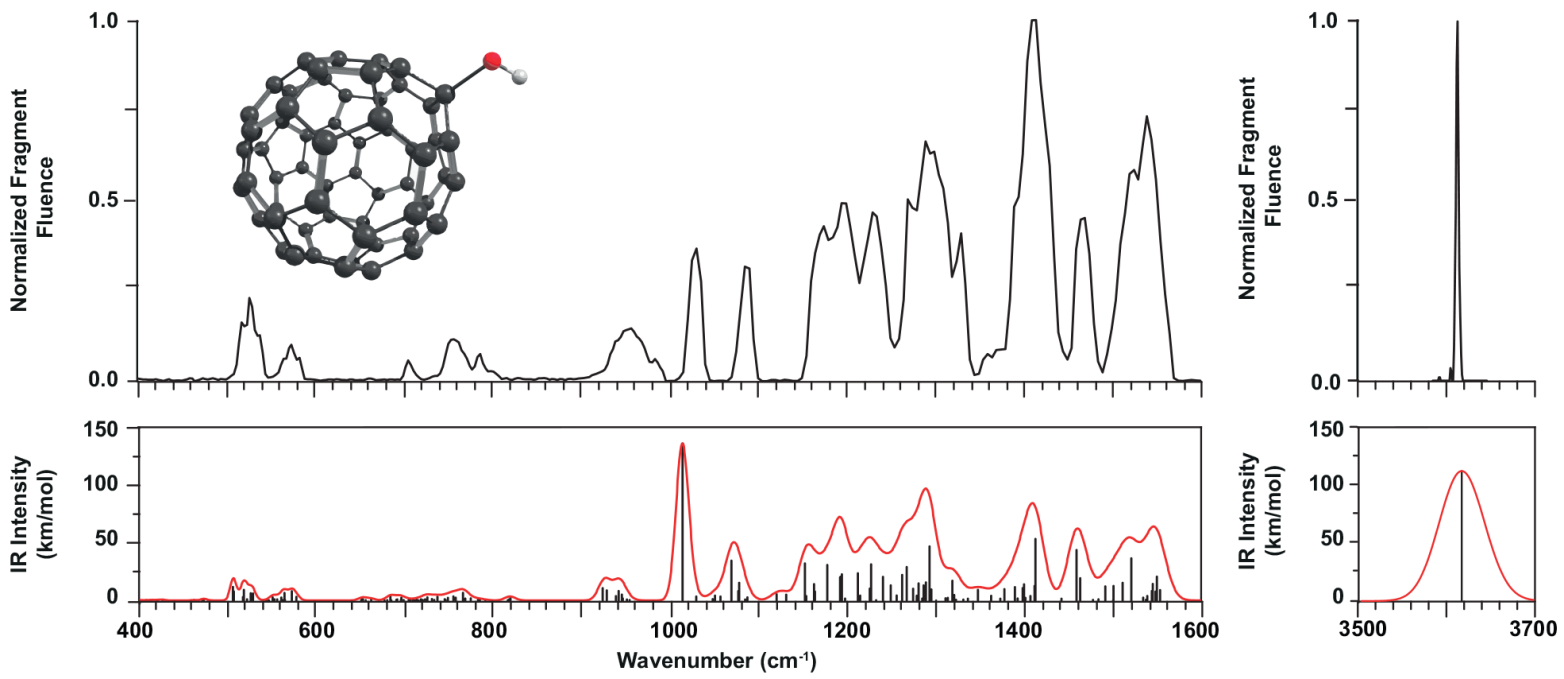
IRMPD spectrum of C_60_OH^+^ (top) compared with the theoretical spectrum computed at the B3LYP/6-311+G(d,p) level. Frequency scaling factors used are 0.955 for the hydrogen stretch range and 0.967 for the 400–1600 cm^–1^ range. The relative intensities in the fingerprint region and in the 3 μm part of the experimental spectrum are unrelated; they have both been normalized to 1.

The IRMPD spectrum of C_60_OH^+^ is shown in Figure 4. As for the radical cation, as well as protonated C_60_,^19^ the spectrum has its strongest bands in the 1000–1600 cm^–1^ range, roughly characterized as CC stretching modes, and weaker features at lower wavenumbers. In the 3500–3700 cm^–1^ range, we detected a single, very significant band using the OPO laser. Since this band at 3613 cm^–1^ can only correspond to an OH stretch vibration, it is immediately clear that protonation must occur on the O atom and not on one of the C atoms. This then also leads to the conclusion that there is only one relevant isomer of C_60_OH^+^ to be considered, reducing greatly the computational effort needed to interpret the spectrum. Comparing the band position of the OH stretch to those of other ionized hydroxyl-substituted aromatic molecules, one sees that the band in C_60_OH^+^ appears shifted toward higher frequencies. As an example, ionized phenol (C_6_H_5_OH^+^) has a strong OH stretch measured at 3534 cm^–1^, and the 1- and 2-isomers of ionized naphthol (C_10_H_7_OH^+^) show OH stretch bands around 3580 cm^–1^.^45,46^ We note that C_60_OH^+^ is a closed-shell cation whereas the aromatic enols are radical cations.

Although protonation occurs exclusively at the oxygen atom, multiple rotamers may exist. The DFT calculations established two distinct minima, one where the proton resides above a five-membered ring and one with the proton above a six-membered ring. However, the energies of the two rotamers are identical to within 1 kJ/mol and the computed vibrational spectra are also virtually identical (see Figure S2), so that we shall further ignore this distinction. The computed spectrum of C_60_OH^+^ reproduces the experimental spectrum closely (Figure 4), allowing for an assignment of the vibrational bands. Between 1100 and 1600 cm^–1^, bands have predominantly CC stretching character, with a few bands showing in addition significant OH bending character. Toward longer wavelengths, there are weaker bands associated mostly with breathing modes and ring deformations. A particularly significant band for this species is the CO stretch mode near 1000 cm^–1^; its relative intensity is significant, although it appears to be overestimated in the computed spectrum. In the 3 μm range, the computed position of the OH stretch band reproduces the experimental value for when a 0.955 scaling factor is used, as recommended for this wavelength range and functional/basis set. The observed fwhm of the OH stretch band is about 3 cm^–1^; the width shown in the computed spectrum is arbitrary and results from the fractional bandwidth used to better match the 6–25 μm wavelength section of the spectrum.

### Astronomical Implications

Given the cosmic abundance of C_60_, fullerene derivatives are likely present to some extent in inter- and circumstellar environments. It has been contemplated by Kroto that exohedral complexes of C_60_ with abundant interstellar species, such as atomic oxygen, are among the most likely derivatives of buckminsterfullerene.^14^ We explore this possibility by comparing the vibrational spectra of ionized fullerene derivatives to the emission spectrum of an astronomical object where the fullerene presence is known.

**Figure 5:**
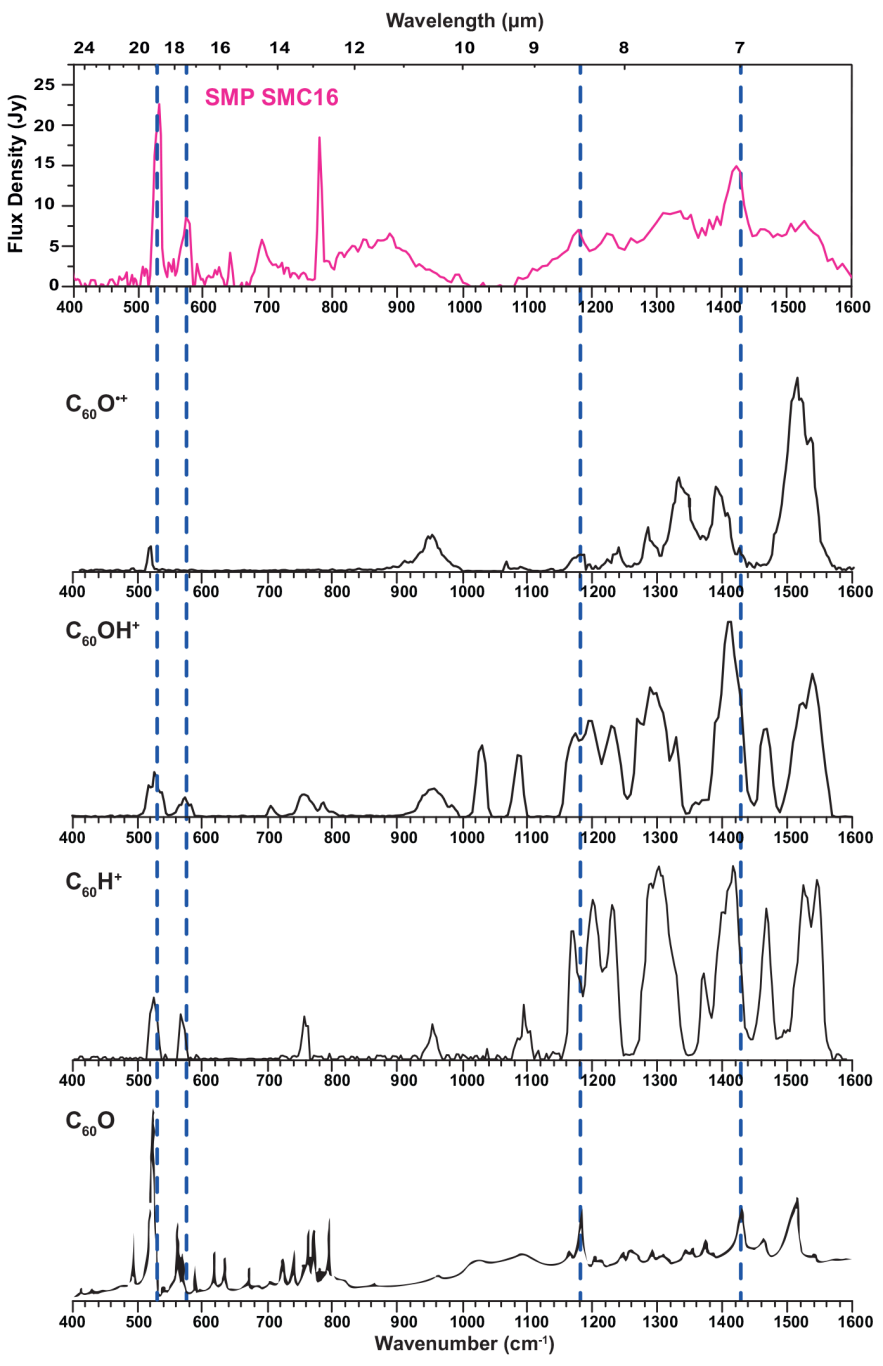
Comparison of the IR spectra of C_60_O^+^, protonated C_60_O, protonated C_60_, and neutral C_60_O. Spectra of ionized species are measured by IRMPD spectroscopy. The spectrum of protonated C_60_ is reproduced with permission from ref (19). Copyright 2019, Springer Nature Limited. The FTIR absorption spectrum of a thin film of neutral C_60_O is reproduced with permission from ref (31). Copyright 1994, American Chemical Society. Dashed lines indicate the vibrational frequencies of neutral C_60_. The experimental spectra are compared to the emission spectrum of the SMP SMC16 nebula, reproduced with permission from ref (47). Copyright 2012, American Astronomical Society.

In Figure 5, the emission spectrum from a nebula that was previously established to be rich in fullerenes^47^ is compared to the experimental spectra of ionized and neutral C_60_ analogues: C_60_O^+^, C_60_OH^+^, C_60_H^+^, and neutral C_60_O. In the SMP SMC16 planetary nebula, the bands of neutral C_60_ peaks are clearly observed, as indicated by the dashed lines representing the band positions of the four C_60_ bands. Moreover, PAH traces are notably missing in this source.^47^ Apart from the C_60_ emission from SMP SMC16, most of the emission features at wavelengths longer than 10 μm have been attributed to various species, such as the SiC feature. However, the broad but structured plateau roughly between 6 and 9 μm has not been accounted for. Hydrogenated amorphous carbon (HAC) grains and PAH clusters have been proposed to be responsible for these features.^11,48^ We noticed earlier that this feature resembles the spectral characteristics of protonated C_60_ and C_70_.^19,20^ We suggested that a mixture of these protonated fullerenes, perhaps together with radical cation fullerenes, may give rise to this emission feature. Here, we add the newly recorded spectra of the oxidized fullerene analogues to the comparison.

Because of the symmetry breaking upon addition of O and OH to the neutral fullerene, we see rich vibrational spectra in all fullerene analogues, similar to what is observed upon protonation. The most striking difference between neutral and ionized C_60_O species is that, in the latter, the highest intensity peaks are featured in the high-wavenumber range (>1000 cm^–1^), whereas the low-wavenumber part of the spectra is quite sparse, showing only peaks with low intensity. For neutral C_60_O, this situation is reversed: the strongest bands are are below 1000 cm^–1^. This observation is confirmed by the computed spectra and is, moreover, similar to the well-known spectral differences between neutral and ionized PAHs.^49−52^ As compared to C_60_H^+^, the appearance of the spectrum of C_60_OH^+^ is quite similar, although there are slight shifts between the two species in the CC stretch modes. The CO stretch mode near 1000 cm^–1^ is diagnostic for C_60_OH^+^. The 1370 cm^–1^ band in C_60_H^+^ is suppressed in the C_60_OH^+^ spectrum.

The strong bands in the 6–9 μm range of C_60_O^+^ and C_60_H^+^ fall within the astronomically observed envelope of the plateau feature. Moreover, other bands in the C_60_O^+^ spectrum do not conflict with the astronomical spectrum, making a contribution from C_60_O^+^ plausible. In the high-wavenumber part of the spectrum, the C_60_OH^+^ features also fall within the astronomical envelope, but the significant CO stretch mode just above 1000 cm^–1^ is not observed. Although the astronomical spectrum shows a weak but distinct feature just below 1000 cm^–1^, this difference in band position appears too large to speculate on the abundance of C_60_OH^+^ in this particular source.

The well-known C_60_ bands at 17.3 and 18.9 μm are broadened in the astronomical spectrum. All fullerene derivatives shown in Figure 5 possess one or two bands at or very close to these positions, such that we speculate that their combined contribution could give rise to the broadening observed. Furthermore, all species show a distinct feature just below 1100 cm^–1^ that coincides with the tail of the plateau in the astronomical spectrum. In addition, all species show absorption around 950 cm^–1^, which coincides with astronomical emission in the blue wing of the SiC feature. The current set of laboratory infrared spectra for these fullerene derivatives warrants future comparison with other astronomical sources.

## Conclusion

We reported the first IR spectra for C_60_OH^+^ and C_60_O^+^, recorded through IRMPD spectroscopy on the gaseous, mass-selected ions. Comparison of the experimental results to DFT calculations enabled us to establish that C_60_O^+^ is formed as the [5,6] annulene isomer, in contrast to what has been reported for neutral C_60_O, which was established to possess the [6,6] epoxide isomeric form. Both in the neutral and in the radical cation, high-level quantum-chemical calculations predict both isomers to be very close in energy. We provide a preliminary comparison of our experimental spectra with an astronomical emission spectrum from the SMP SMC16 planetary nebula and contemplate the possible interstellar abundance of oxidized fullerenes, as hypothesized by Kroto many years ago.

## Supporting Information Available

Additional computed spectra obtained using alternative DFT functionals for the two isomers of C_60_O^+^, computed spectra for the two rotamers of C_60_OH^+^, comparison of computed and experimental band centers for C_60_O^+^, optimized geometries for [5,6] C_60_O^+^, [6,6] C_60_O^+^, and C_60_OH^+^ (PDF)
