# Supplementary material for: Laboratory IR Spectra of the Ionic Oxidized Fullerenes C60O+ and C60OH+
Source: J Phys Chem A. 2022 May 9;126(19):2928–35. doi: 10.1021/acs.jpca.2c01329 (PMC9125688; doi:10.1021/acs.jpca.2c01329)
Supplement: Supplementary file 1 — jp2c01329_si_001.pdf [file jp2c01329_si_001.pdf]

Laboratory IR Spectra of the Ionic Oxidized Fullerenes, $C_{60}O^+$ and $C_{60}OH^+$ Supporting Information

Julianna Palotás,[†] Jonathan Martens,[†] Giel Berden,[†] and Jos Oomens[*,†,‡]

†*Radboud University, Institute for Molecules and Materials, FELIX Laboratory, Toernooiveld 7, 6525ED Nijmegen, The Netherlands*

‡*van 't Hoff Institute for Molecular Sciences, University of Amsterdam, Science Park 904, 1098XH Amsterdam, The Netherlands*

E-mail: j.oomens@science.ru.nl

Calculated IR spectra of $C_{60}O^+$

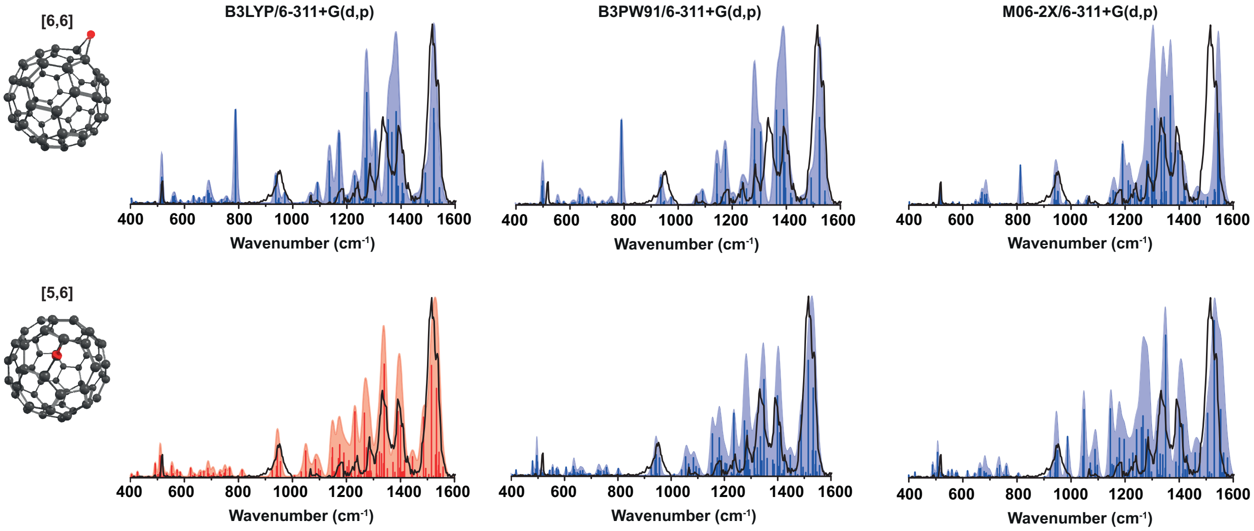

Figure S1: Theoretical spectrum of the isomers of $C_{60}O^+$ calculated at the B3LYP/6-311+G(d,p) (scaled by 0.967), B3PW91/6-311+G(d,p) (scaled by 0.957) and M06-2X/6-311+G(d,p) (scaled by 0.952) levels.

Calculated IR spectra of $C_{60}OH^+$

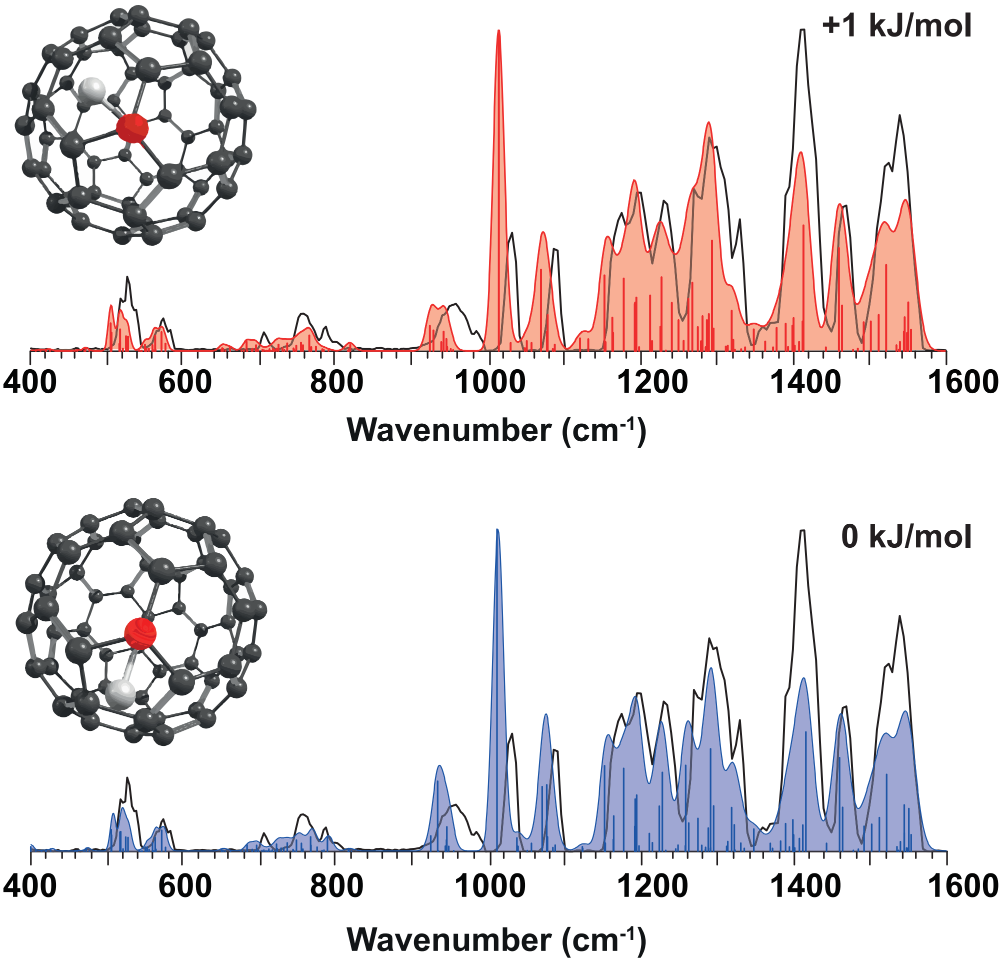

Figure S2: Theoretical spectra of the two rotamers of $C_{60}OH^+$ compared to the experimental spectrum of $C_{60}OH^+$ (black curve). The spectra are calculated at the B3LYP/6-311+G(d,p) level with harmonic frequencies scaled by 0.967. The proton is either above the 6-membered ring (red shade) or above the 5-membered ring (blue shade). The energy of the two rotamers is identical within 1 kJ/mol and the two computed spectra are virtually identical; the OH stretch band (not shown) coincides within 1.5 cm^{-1}.

Experiment versus theory spectral comparison

Table S1: Band centers in cm^{-1} from the experimental spectrum and from the convolved theoretical spectra of isomers of $C_{60}O^{+}$. Deviations between experimental and calculated values (Δ) and the root-mean-square deviations (RMSD) are also listed. Harmonic frequencies are calculated at the B3LYP/6-311+G(d,p) level and scaled by 0.967.

**Exp**		**Computed**		
	**[5,6]**	Δ	**[6,6]**	Δ
-	492.2	-	-	-
519	510.6	8.4	514.4	4.6
-	554.1	-	559.9	-
-	623.7	-	632.4	-
-	688.5	-	689.5	-
-	750.4	-	754.3	-
-	816.1	-	788.1	-
952	944.8	7.2	937	15.0
-	-	-	969.9	-
1067	1049.2	17.8	1064.7	2.3
1088	1087.9	0.1	1090.8	−2.8
1136	1148.8	−12.8	1135.3	0.7
1181	1173.0	8.0	1170.1	10.9
1240	1231.0	9.0	1229.1	10.9
1285	1270.6	14.4	1272.6	12.4
1333	1337.4	−4.4	1305.5	27.5
1390	1395.4	−5.4	1381.8	8.2
-	1444.7	-	-	-
-	1488.2	-	-	-
1516	1527.9	−11.9	1521.1	−5.1
**RMSD**		10.21		11.68

Optimized geometries

Table S2: Atom positions in Å for isomer [5,6] of $C_{60}O^{+}$, calculated with B3LYP/6-311+G(d,p).

Atom	X (Å)	Y (Å)	Z (Å)
C	-1.688993	3.094492	0.319591
C	-2.110011	2.335823	1.482575
C	-1.018614	2.314246	2.411267
C	0.092821	3.057301	1.840674
C	-0.321132	3.525939	0.532535
C	0.582506	3.508759	-0.527088
C	1.942326	3.046494	-0.313922
C	2.340468	2.612851	0.947507
C	1.391545	2.615349	2.046803
C	1.630759	1.427823	2.848753
C	0.558967	0.72547	3.400145
C	-0.796436	1.166274	3.163325
C	-1.650208	0.000182	2.995361
C	-2.729193	0.000086	2.098813
C	-2.993853	1.2518	1.35678
C	-3.609933	1.085974	0.078449
C	-3.609866	-1.086159	0.078611
C	-2.99377	-1.251754	1.356955
C	-2.109864	-2.33571	1.48291
C	-1.018477	-2.313935	2.411594
C	-0.796363	-1.165834	3.163485
C	0.559007	-0.72492	3.400245

Continued on next page

Table S2 – *Continued from previous page*

Atom	X (Å)	Y (Å)	Z (Å)
C	1.630844	-1.427296	2.848954
C	2.727053	-0.701851	2.249316
C	2.727011	0.702343	2.249212
C	3.169595	1.429478	1.077723
C	3.571312	0.728416	-0.058006
C	3.159516	1.177583	-1.367737
C	2.36002	2.31132	-1.495619
C	1.255358	2.30896	-2.430535
C	0.988843	1.174892	-3.198511
C	1.835775	-0.000183	-3.079594
C	2.893083	-0.000085	-2.181374
C	3.159593	-1.177615	-1.367568
C	3.57136	-0.728227	-0.057898
C	3.169687	-1.429141	1.077932
C	2.340625	-2.612577	0.94789
C	1.3917	-2.614961	2.047182
C	0.093003	-3.057018	1.841117
C	-0.320921	-3.525876	0.533049
C	-1.688805	-3.094535	0.320038
C	-2.089354	-2.6727	-0.944328
C	-3.013033	-1.585233	-1.072845
C	-2.558897	-0.717417	-2.174793
C	-1.433365	-1.414722	-2.789056
C	-1.146291	-2.616006	-2.031229

Continued on next page

Table S2 – *Continued from previous page*

Atom	X (Å)	Y (Å)	Z (Å)
C	0.159447	-3.048688	-1.838656
C	0.582717	-3.508806	-0.526575
C	1.94251	-3.046435	-0.313477
C	2.360163	-2.311415	-1.495283
C	1.255499	-2.30926	-2.430208
C	0.98892	-1.175325	-3.198348
C	-0.372013	-0.724549	-3.370183
C	-0.372054	0.724012	-3.37028
C	-1.433454	1.414214	-2.789254
C	-2.558941	0.716945	-2.174901
C	-3.013137	1.584904	-1.073072
C	-2.089514	2.672436	-0.944714
C	-1.146448	2.61563	-2.031604
C	0.159263	3.048419	-1.839098
O	-4.431267	-0.000137	-0.092272

Table S3: Atom positions in Å for isomer [6,6] of $C_{60}O^+$, calculated with B3LYP/6-311+G(d,p).

Atom	X (Å)	Y (Å)	Z (Å)
C	0.61449	2.606814	-2.299999
C	1.054301	1.430329	-3.014727
C	-0.113737	0.704812	-3.446785

Continued on next page

Table S3 – *Continued from previous page*

Atom	X (Å)	Y (Å)	Z (Å)
C	-1.287088	1.433516	-3.021037
C	-0.838017	2.611721	-2.301733
C	-1.529159	3.03742	-1.173984
C	-2.700341	2.306724	-0.7232
C	-3.134136	1.176749	-1.419646
C	-2.408224	0.731042	-2.583531
C	-2.408298	-0.7305	-2.583631
C	-1.287234	-1.433018	-3.021226
C	-0.113804	-0.704358	-3.446875
C	1.054155	-1.430029	-3.014903
C	2.179762	-0.730882	-2.568363
C	2.179836	0.731026	-2.568277
C	2.931665	1.193571	-1.445588
C	3.511905	-0.000106	-0.762684
C	2.931541	-1.193638	-1.445739
C	2.489662	-2.302152	-0.731243
C	1.315075	-3.029758	-1.174146
C	0.614235	-2.606572	-2.300329
C	-0.838272	-2.611352	-2.302071
C	-1.529456	-3.037135	-1.174376
C	-2.700571	-2.306395	-0.723501
C	-3.134255	-1.176292	-1.419798
C	-3.583987	0.000203	-0.693341
C	-3.583984	0.000113	0.693356

Continued on next page

Table S3 – *Continued from previous page*

Atom	X (Å)	Y (Å)	Z (Å)
C	-3.134128	1.176565	1.419809
C	-2.700337	2.306631	0.723507
C	-1.529154	3.03727	1.17438
C	-0.838007	2.611426	2.302072
C	-1.287076	1.433129	3.021227
C	-2.408213	0.73071	2.583635
C	-2.408287	-0.730833	2.583548
C	-3.134251	-1.176475	1.419661
C	-2.700569	-2.306489	0.723216
C	-1.529451	-3.037285	1.173992
C	-0.801109	-3.485018	-0.000222
C	0.589649	-3.478282	-0.000225
C	1.31508	-3.029908	1.173751
C	2.489666	-2.302246	0.730937
C	2.931548	-1.193824	1.445573
C	3.511908	-0.000204	0.762669
C	2.93167	1.193385	1.445729
C	2.179847	0.730696	2.568361
C	2.179774	-0.731212	2.568261
C	1.054168	-1.430416	3.014715
C	0.614245	-2.606868	2.299992
C	-0.838262	-2.611647	2.301739
C	-1.28722	-1.433405	3.021047
C	-0.113789	-0.7048	3.446786

Continued on next page

Table S3 – *Continued from previous page*

Atom	X (Å)	Y (Å)	Z (Å)
C	-0.113723	0.704369	3.446876
C	1.054314	1.429942	3.014906
C	0.6145	2.606519	2.300331
C	1.315376	3.02964	1.174142
C	2.489897	2.301936	0.731239
C	2.489893	2.30203	-0.730954
C	1.315371	3.029791	-1.173759
C	0.589985	3.478227	0.000222
C	-0.800773	3.485089	0.000226
O	4.703546	-0.000222	-0.00001

Table S4: Atom positions in Å for $C_{60}OH^+$, calculated with B3LYP/6-311+G(d,p).

Atom	X (Å)	Y (Å)	Z (Å)
C	1.04182	-2.160189	-2.570824
C	1.621599	-2.754698	-1.389062
C	0.565492	-3.411451	-0.652306
C	-0.675809	-3.229544	-1.381184
C	-0.381886	-2.446839	-2.566776
C	-1.28665	-1.485704	-3.009442
C	-2.517086	-1.264591	-2.28047
C	-2.806061	-2.021113	-1.145294
C	-1.86342	-3.021285	-0.685772

Continued on next page

Table S4 – *Continued from previous page*

Atom	X (Å)	Y (Å)	Z (Å)
C	-1.863675	-3.001519	0.766724
C	-0.676415	-3.190971	1.468231
C	0.565148	-3.393008	0.745575
C	1.62127	-2.716594	1.464831
C	2.637844	-2.061205	0.75852
C	2.637314	-2.07949	-0.699939
C	3.12665	-0.828023	-1.174274
C	3.685835	-0.001197	-0.001797
C	3.128258	-0.79661	1.199089
C	2.556433	-0.185024	2.295973
C	1.496547	-0.837814	3.03086
C	1.04029	-2.089625	2.629739
C	-0.383175	-2.376157	2.632388
C	-1.288216	-1.403207	3.048142
C	-2.518261	-1.201964	2.31275
C	-2.806371	-1.989026	1.19824
C	-3.384181	-1.379142	0.017874
C	-3.66467	-0.010623	-0.000766
C	-3.37463	0.772003	-1.183872
C	-2.812663	0.160106	-2.301681
C	-1.752646	0.820267	-3.027839
C	-1.302354	2.077873	-2.621619
C	-1.893746	2.720353	-1.460452
C	-2.904499	2.078283	-0.755536

Continued on next page

Table S4 – *Continued from previous page*

Atom	X (Å)	Y (Å)	Z (Å)
C	-2.904911	2.097963	0.697424
C	-3.375359	0.803821	1.160866
C	-2.814226	0.222568	2.295285
C	-1.754402	0.902326	3.003566
C	-0.809101	-0.099915	3.474673
C	0.549126	0.177744	3.465669
C	1.023328	1.467511	2.997232
C	2.237629	1.245728	2.259949
C	2.534152	2.026964	1.130746
C	3.081524	1.400956	-0.019268
C	2.536585	1.996096	-1.184342
C	1.592557	3.026064	-0.763816
C	1.591787	3.046193	0.682374
C	0.403588	3.236453	1.371107
C	0.111002	2.428025	2.54866
C	-1.304358	2.148561	2.563879
C	-1.894963	2.75925	1.385365
C	-0.837654	3.431452	0.650083
C	-0.836963	3.412326	-0.742965
C	0.404778	3.197865	-1.45763
C	0.112862	2.357585	-2.613457
C	1.025437	1.385614	-3.035118
C	2.240344	1.183815	-2.291892
C	2.557651	-0.246454	-2.2874

Continued on next page

Table S4 – *Continued from previous page*

Atom	X (Å)	Y (Å)	Z (Å)
C	1.497825	-0.919219	-3.003699
C	0.550949	0.083727	-3.467953
C	-0.807192	-0.194317	-3.470972
O	5.105486	0.042084	-0.094538
H	5.475633	0.437506	0.705913
